# Recognition of FT-IR Data Cuscutae Semen, Japanese Dodder, and Sinapis Semen Using Discrete Wavelet Transformation and RBF Networks

**DOI:** 10.1155/2013/853483

**Published:** 2013-10-24

**Authors:** Tao Hu, Xuexiang Weng, Lishan Xu, Cungui Cheng, Peng Yu

**Affiliations:** ^1^Faculty of Life Science and Chemical Engineering, Huaiyin Institute of Technology, Huaian 223003, China; ^2^National Special Superfine Powder Engineering Center, Nanjing University of Science and Technology, Nanjing 210094, China; ^3^College of Chemistry and Life Science, Zhejiang Normal University, Jinhua 321004, China; ^4^Zhejiang Jiaxing Entry-Exit and Inspection and Quarantine Bureau, Jiaxing 314000, China

## Abstract

Horizontal attenuation total reflection Fourier transformation infrared spectroscopy (HATR-FT-IR) studies on cuscutae semen and its confusable varieties Japanese dodder and sinapis semen combined with discrete wavelet transformation (DWT) and radial basis function (RBF) neural networks have been conducted in order to classify them. DWT is used to decompose the FT-IRs of cuscutae semen, Japanese dodder, and sinapis semen. Two main scales are selected as the feature extracting space in the DWT domain. According to the distribution of cuscutae semen, Japanese dodder, and sinapis semen's FT-IRs, three feature regions are determined at detail 3, and two feature regions are determined at detail 4 by selecting two scales in the DWT domain. Thus five feature parameters form the feature vector. The feature vector is input to the RBF neural networks to train so as to accurately classify the cuscutae semen, Japanese dodder, and sinapis semen. 120 sets of FT-IR data are used to train and test the proposed method, where 60 sets of data are used to train samples, and another 60 sets of FT-IR data are used to test samples. Experimental results show that the accurate recognition rate of cuscutae semen, Japanese dodder, and sinapis semen is average of 100.00%, 98.33%, and 100.00%, respectively, following the proposed method.

## 1. Introduction

At present in the medical field, the biggest achievement of the human beings is western medicine and traditional Chinese medicine (TCM). With a history of 2000 to 3000 years, TCM has formed a unique system to diagnose and cure illness. Herbal medicines have gained increasing attention worldwide for the treatment of chronic diseases because of their effectiveness and small side effects [1, 2]. Cuscutae semen is a kind of TCM, which has been known for the treatment of diseases for a long time. The main effect of cuscutae semen is to replenish the kidney essence or “jing” in order to support healthy sexual and reproductive functioning. Through the mother-son relationship between kidney and liver, it also benefits the eyes, tendons, and other physiological functions related to the kidneys and liver [3]. In order to ensure the safety of clinical medicine, the quality of the TCM should be scientific evaluation. However, the complicated constituents contained in TCMs create analytical problems in terms of quality control [4].

Fourier transform infrared spectroscopy (FT-IR) can get nearly all of material information about complex systems, so it is successful using FT-IR analysis in different families and genera. As two TCM samples are sibling species, they contain similar chemical composition. Therefore, the result is not ideal when FT-IR analysis was only adopted. How to make use of the large amount of data of absorption spectra from complex system for fast qualitative and quantitative analysis effectively and make the information that is buried in the FT-IR overlapping bands and the difference that existed in the infrared absorptions spectra be displayed visually for the identification of those spectra which are similar and complicated have been a goal of analytical chemists [5–7]. The presence of grape seed oil in *Nigella sativa L*. seed oil had been analyzed by Fourier transform infrared spectroscopy. The results had been disposed with some methods of chemometrics [8]. Some analysis of miR-205 and miR-155 expression in the blood of breast cancer patients was to identify and validate circulating microRNAs (miRNAs) in human plasma for use as breast cancer (BC) biomarkers and to analyze their relationship to clinicopathologic features and its preliminary biological function. Functional analysis showed that ectopic expression of miR-205 significantly inhibits cell proliferation and promotes apoptosis. miR-205 was downregulated and miR-155 was upregulated in BC patient serum. miR-155 was positive correlated with clinical stage and ki-67 and negatively correlated with p53 status [9].

Wavelet transformation is a more effective signal processing method than Fourier transform, and the transformed results (wavelet factor) of discrete wavelet transform (DWT) contain more valuable information, which is a relatively effective analysis method in chemometrics. The wavelet transformation is being used in chemistry and its related domains in recent years [10, 11].

An artificial neural network (ANN) is a mathematical model or computational model that is inspired by the structure and/or functional aspects of biological neural networks. ANN, with their remarkable ability to derive meaning from complicated or imprecise data, can be used to extract patterns and detect trends that are too complex to be noticed by either humans or other computer techniques. A trained ANN can be thought of as an “expert” in the category of information it has been given to analyze [12, 13]. The wave near infrared spectroscopy combined with the artificial neural network to establish the sugar juice brix and rotation of quantitative analysis model. The results showed that the artificial neural network can be better used for juice brix and the rapid determination of the optical rotation [14]. Recently, discrete stationary wavelet transformation (DSWT) and probability neural networks have been successfully applied to FT-IR analysis, but few studies have been reported in the FT-IR-DWT-radial basis function (RBF) neural network application to recognition TCM [6, 8]. Therefore, HATR-FT-IR spectroscopy combined with DWT and RBF neural network discrimination method was proposed for the rapid and simple classification of cuscutae semen, Japanese dodder, and sinapis semen making a difficult distinction among them from morphology in this study.

## 2. Experimental

### 2.1. Sample Preparation

Cuscutae semen (Cuscuta seed, or Chinese dodder seed) is the dried and matured seed of *Cuscuta chinensis* Lam. (Convolvulaceae). Japanese dodder (dodder seed) is the dried matured seed of *Cuscuta japonica *Choisy (Convolvulaceae). Sinapis semen is the dried matured seed of *Brassica juncea* (L.) Czern. Et Coss (Cruciferae). All of samples were collected from Jinhua (28°48′N, 119°32′E) of Zhejiang province, Leshan (29°20′N, 103°50′E) of Sichuan province, and Linyi (35°50′N, 118°32′E) of Shandong province, China, in October, 2004, and dried in sunlight, respectively. Eight seed samples are selected randomly for one time per sample. The samples have been grounded to fine powder in agate mortars to about 100 meshes, respectively. 

### 2.2. Spectra Collection

The HATR-FT-IR spectra were collected at a resolution of 2 cm^−1^ scans using a Thermo Electron (Madison, WI, USA) Nexus 670 FT-IR spectrometer with a room temperature deuterated triglycine sulfate (DTGS) detector, and with a single-bounce HATR (Ge) accessory, spectral range 4000–650 cm^−1^, and the cumulative number of scan 64 times; 8.0 mg of predisposed samples was, respectively, placed directly about 3.14 mm^2^ on the center of the Ge crystal of the HATR accessory for measurement. To ensure good contact with the Ge crystal surface, all powder samples were pressed using a pressure tower to provide the same mechanical pressure on all samples. All obtained spectra were autobaseline corrected. No other sample preparation was required. Each species of all samples was measured three times and the averaged spectrum was used for further analysis.

### 2.3. Basic Theory of Cluster Analysis

Cluster analysis, also called segmentation analysis or taxonomy analysis, is a classification technique in common use in biostatistics, which can be applied to many different kinds of data. A meaningful clustering method will depend on choosing appropriate classification parameters and a consistent method to measure the similarity between the data points and result in a parameter to define “similarity” and a method to quantify the similar of two data points. In this respect, it is also crucial to define a way to quantify “how similar” two objects are, which, in more quantitative terms, is equivalent to “how close or far apart” two objects are from each other in terms of their similarity.

Suppose there are *n* objects *A*_1_; *A*_2_; …; *A*_*i*_; …; *A*_*n*_ and *m* attributes (factors) *B*_1_; *B*_2_; …; *B*_*j*_; …; *B*_*m*_. Given a set of *X* = {*X*_1_; *X*_2_; …; *X*_*i*_; …; *X*_*n*_}, where *X*_*i*_ is a vector, that is, *X*_*i*_ = (*X*_*i*1_; *X*_*i*2_; …; *X*_*ij*_), and *X*_*j*_ = (*c*_*ij*_, *a*_*ij*_, *b*_*ij*_, *d*_*ij*_), *i* = 1,2; …, *n*; *j* = 1,2; …, *m*, to be the attribute *B*_*j*_'s preference rating of *i*th object *A*_*i*_. The systematic clustering method presented herein can be summarized as follows. 

Define the normalized attribute preference rating of *X*_*ij*_, and denote it by *X*_*ij*_^~^ as follows:

(1) $$\begin{array}{c} X_{ij}^{\sim }=\left[\frac{\left(c_{ij}-c_{j}^{*}\right)}{t_{j}^{*}},\frac{\left(a_{ij}-c_{j}^{*}\right)}{t_{j}^{*}},\frac{\left(b_{ij}-c_{j}^{*}\right)}{t_{j}^{*}},\frac{\left(d_{ij}-c_{j}^{*}\right)}{t_{j}^{*}}\right], \end{array}$$

where

(2) $$\begin{array}{c} t_{j}^{*}=d_{j}^{*}-c_{j}^{*},\;d_{j}^{*}=\underset{i}{\max}\left\{d_{ij}\right\},\;\;c_{j}^{*}=\underset{i}{\min}\left\{c_{ij}\right\}. \end{array}$$

According to the distance function between two trapezoidal fuzzy numbers, define the fuzzy compatibility relation *R* as follows:

(3) $$\begin{array}{c} R\left(X_{i},X_{k}\right)=1-\eta \left[\overset{m}{\underset{j=1}{\sum }}d_{2}\left(X_{ij}^{\sim },X_{kj}^{\sim }\right)\right], \end{array}$$

where *η* is the inverse value of the largest distance in *X*, that is,

(4) $$\begin{array}{c} \eta ={\left\{\underset{i,k}{\max}\left\{\overset{m}{\underset{j=1}{\sum }}d_{2}(X_{ij}^{\sim },X_{kj}^{\sim })\right\}\right\}}^{-1}. \end{array}$$

Find the transitive closure Rt by utilizing algorithm A. Then, find all the feasible clusters by taking suitable *λ* value.

Suitable *λ* value (*λ* ∈ [0,1]), variable from 1 to 0, and the obtain series cluster could be taken based on the fuzzy compatibility relation *R* stated as above.

A cluster validity index *L*, which modified the compactness and separation validity function, can be used to determine the best number of clusters. Define

(5) $$\begin{array}{c} L=\frac{T}{n\times d_{\min}^{2}}, \end{array}$$

where

(6) $$\begin{array}{c} T=\overset{h}{\underset{r=1}{\sum }}\overset{n}{\underset{i=1}{\sum }}u_{ir}d_{2}^{2}\left(X_{i}^{\sim },V_{r}^{\sim }\right),\\ d_{\min}^{2}=\underset{q,r}{\min}d_{2}^{2}\left(V_{q}^{\sim },V_{r}^{\sim }\right),\\ u_{ir}=\{\begin{array}{cc} 1, & \text{if}\;\;A_{i}\in C_{r},\\ 0, & \text{if}\;\;A_{i}\notin C_{r}, \end{array}\\ d_{2}^{2}\left(X_{i}^{\sim },V_{r}^{\sim }\right)=\overset{m}{\underset{j=1}{\sum }}d_{2}^{2}\left(X_{ij}^{\sim },V_{rj}^{\sim }\right),\\ d_{2}^{2}\left(V_{q}^{\sim },V_{r}^{\sim }\right)=\overset{m}{\underset{j=1}{\sum }}d_{2}^{2}\left(V_{qj}^{\sim },V_{rj}^{\sim }\right), \end{array}$$

where *A*_*i*_ is the *i*th object, *h* is the number of clusters, *n* is the number of objects, *m* is the number of attributes, *X*_*i*_^~^ is the normalized attribute preference rating vector of object *A*_*i*_ versus all attributes, that is, *X*_*i*_^~^ = (*X*_*i*1_^~^,…, *X*_*ij*_^~^,…, *X*_*im*_^~^), *V*_*r*_^~^ is the normalized centroid vector of cluster *C*_*r*_, that is, *V*_*r*_^~^ = (*V*_*r*1_^~^,…, *V*_*rj*_^~^,…, *V*_*rm*_^~^), *C*_*r*_ is the *r*th cluster, *d*_2_^2^(*X*_*ij*_^~^, *V*_*rj*_^~^) is Chen's modified geometrical distance square between *X*_*ij*_^~^ and *V*_*rj*_^~^ with parameter *p* = 2, *d*_2_^2^(*V*_*qj*_^~^, *V*_*rj*_^~^) is Chen's modified geometrical distance square between *V*_*qj*_^~^ and *V*_*rj*_^~^ with parameter *p* = 2.

Since the more separate the clusters, the larger *d*_min⁡_^2^, and the smaller *L*. Thus, the smallest *L* indeed indicates a valid optimal clustering. After calculating the *L* value of various clusters obtained, the best number of clusters and the objects belonging to each cluster can be obtained [15–17].

### 2.4. Basic Theory of DWT

DWT is a wavelet transformation that the wavelets are discretely sampled in numerical analysis and functional analysis. It is used for signal coding to represent a discrete signal in a more redundant form and often as a preconditioning for data compression for it has a key advantage over that it captures both frequency and location information over Fourier transforms. DWT is originated from the discretization of continuous wavelet transformation (CWT), and the common discretization is dyadic. 

 The function of DWT is accordingly expressed as

(7) $$\begin{array}{c} W_{\text{DWT}}\left(j,k\right)=\frac{1}{\sqrt{2^{j}}}\overset{\infty }{\underset{-\infty }{\int }}f\left(t\right)\Psi^{*}\left(\frac{t-2^{j}k}{2^{j}}\right)dt. \end{array}$$

The original signal *f*(*t*) passes through two complementary filters and emerges as low frequency and high frequency signals. With successive approximations being decomposed in turn, the decomposition process can be iterated, so that a signal can be broken down into many lower-resolution components [11]. 

### 2.5. Basic Theory of RBF Networks

**Figure 1:**
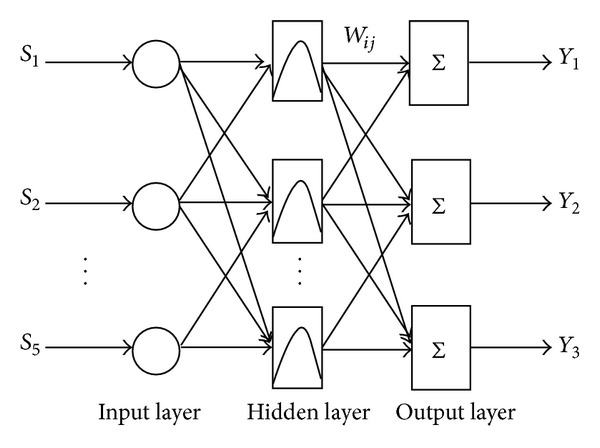
Structure of RBF neural network.

RBF neural network can extend or preprocess the input vector to the high-dimensional space. It not only has good generalization ability and also avoids the complex computation as back-propagation neural network. Therefore we can achieve the rapid learning of neural network. In this paper, we aim at the classification and identification of three kinds of plant seeds (cuscutae semen, Japanese dodder and sinapis semen). Five feature parameters are used as input vector, thus the input layer of the network needs five neurons. Therefore the RBF neural network has five input neural units and three output neural units. Structure of RBF neural network is shown in Figure 1.

The first layer is the input layer, it introduces eigenvector {*S*_1_, *S*_2_,…, *S*_5_} into the network. The second layer is hidden layer, which is fully connected with the input layer (weight value = 1). Its role is equal to a conversion to the input modes, which transforms low-dimensional model input data to the high-dimensional space, to be in favor of the output layer's classification and recognition. Hidden layer node selects basis function as a transfer function [6].

### 2.6. Data Analysis

HATR-FT-IR of all the samples can be obtained by determination. According to the absorbance value characteristic of absorption peak, we can make the cluster analysis to the data, which are carried out by the Ward clustering algorithm. Then, MATLAB V6.5 (Mathworks, USA) software is used to make wavelet transform to analyze the data further. Using Daubechies wavelet, which has a good detection capability of the signal singularity, as the analysis wavelet, one-dimensional DWT is done to the FT-IR spectra of samples under different scales. Then, the differences of HATR-FT-IR spectra of the samples in various scales are compared. We choose two representative details to extract features of samples and then use RBF neural network to identify them. In the experiment, we make one-dimensional DWT to the HATR-FT-IR spectra of the samples (they are decomposed into 5 scales). We choose two scales (3 and 4) as the scales to extract the feature vector. The number of training samples and testing samples, which come from three locations, was 180, respectively. 20 samples were randomly selected in each location per species. The selected character variables were used for RBF neural network training and identification.

## 3. Results and Discussion

### 3.1. Spectra Investigation

**Figure 2:**
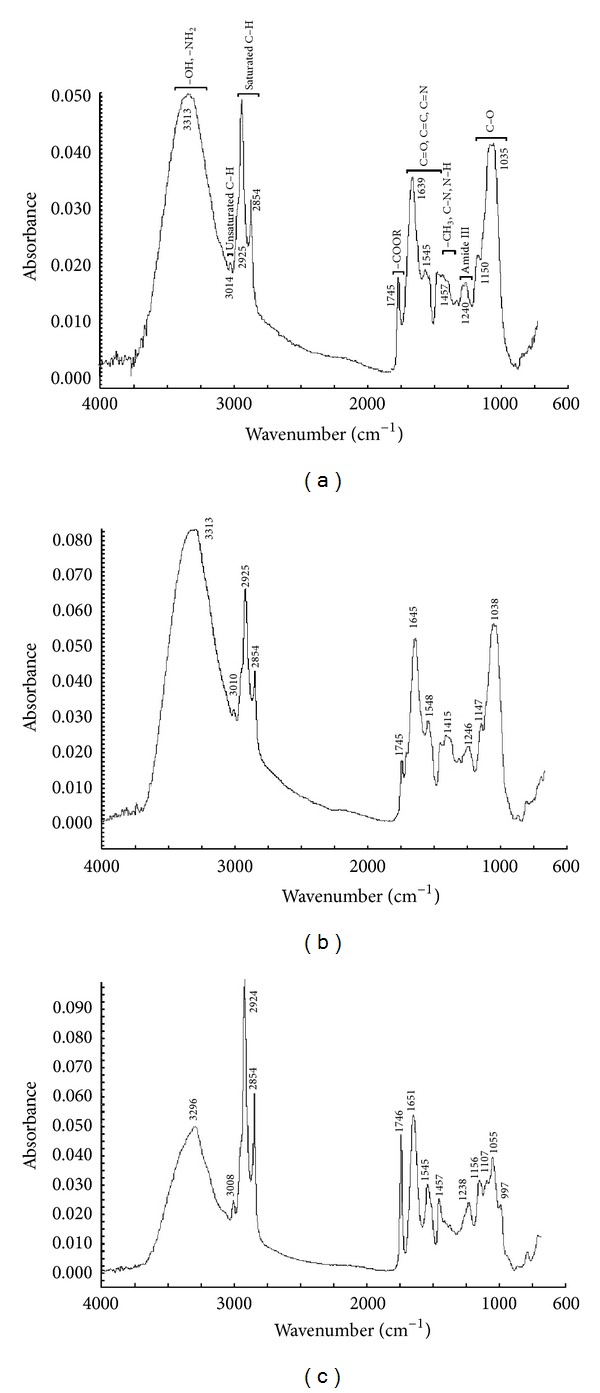
FT-IR spectra of (a) cuscutae semen; (b) Japanese dodder; and (c) sinapis semen.

Figure 2 shows the typical HATR-FT-IR spectra of cuscutae semen, Japanese dodder, and sinapis semen. 

From Figure 2, we notice that the cuscutae semen, Japanese dodder and sinapis semen generated large numbers of sharp peaks in the FT-IR spectra region (4000–650 cm^−1^), which indicates that the seeds have a rich chemical composition. Several absorption regions were identified, and the band assignments are labeled in Figure 2. Absorption bands located around 3400 cm^−1^ correspond to O–H and N–H stretching vibrations that mainly occur from carbohydrates and protein mainly. The bands around 3010 cm^−1^ represent unsaturated C–H stretching vibrations that are mainly caused by unsaturated compounds and unsaturated fatty acid ester. The bands around 2925 cm^−1^ and 2854 cm^−1^ represent saturated C–H stretching vibrations that are mainly caused by lipid, carbohydrates, and saturated C–H in other compounds. Absorption raised from C–H bending modes was located around 1200 cm^−1^ to 1500 cm^−1^ but overlap with other absorption bands within this region. Three protein absorption bands located around 1650 cm^−1^ (C=O), 1456 cm^−1^ (N–H), and 1240 cm^−1^ (C–N) were assigned as amide I, II, and III bands, respectively. Absorption bands around 1745 cm^−1^ correspond to isolated carbonyl group (COOR), indicating ester-containing compounds commonly found in membrane lipid and cell wall pectin. Bands around 1035 cm^−1^ and 1150 cm^−1^ in the “fingerprint” region indicate several modes such as C–H bending vibration or C–O or C–C or P–O stretching vibration.

The cuscutae semen and Japanese dodder are similar in absorption peaks in the FT-IR spectra because they belong to the sibling plant seeds. They contain similar chemical composition like hydroxy of cellulose (seed coat), carbohydrates, protein, phytosterol, flavonoids, alkaloids, and so forth, and their FT-IR absorption are quite similar. The FT-IR spectra of the cuscutae semen and Japanese dodder from the different plant seeds have very closed absorbance and are difficult to distinguish by experience. So, we use other methods for further classification.

### 3.2. Cluster Analysis

**Figure 3:**
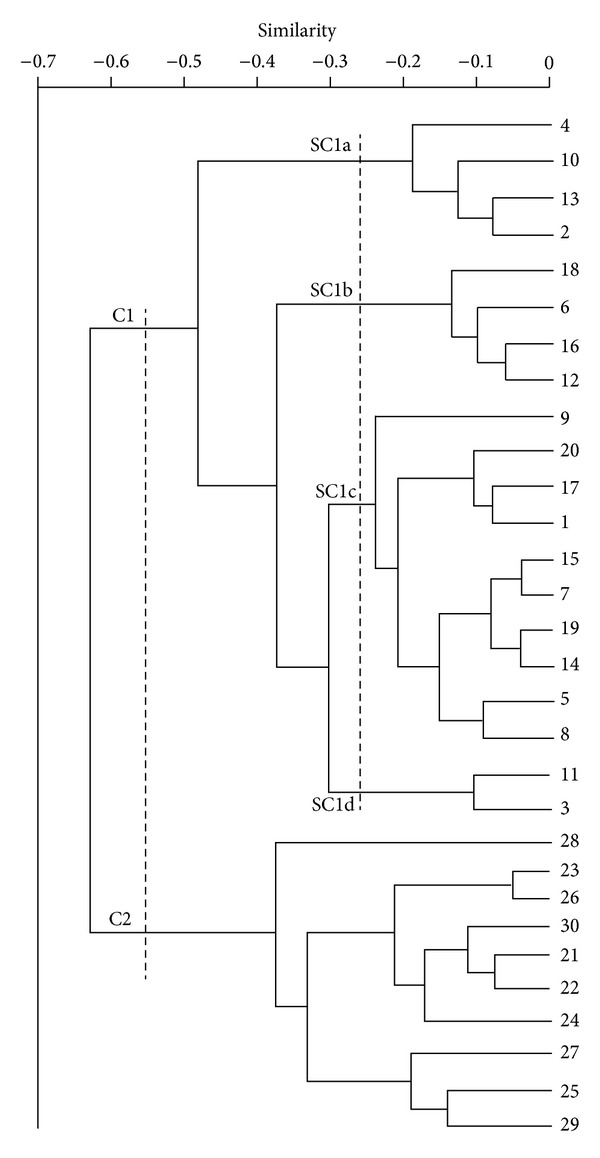
Dendrogram obtained by hierarchical cluster analysis of 30 samples based on FT-IR spectra.

In this paper, 10 samples of each species are randomly selected to make cluster analysis. We have selected 17 absorption peaks in the range of the 4000~650 cm^−1^, and then the absorption peaks which we select are tested by cluster analysis. When they are of different families, the results are satisfactory, but when they are of sibling samples, the results are not satisfactory. The dendrogram is shown in Figure 3.

Samples a1–a10 are Cuscutae semen; samples b1–b10 are Japanese dodder; samples c1–c10 are Sinapis semen.

From Figure 3, 30 samples are divided into two families by the dendrogram: cluster 1 (C1) is a rather loose cluster which includes sample a1–a10 in cuscutae semen and sample b1–b10 in Japanese dodder; cluster 2 (C2) comprises all the remaining samples in sinapis semen. On closer inspection, C1 can be seen to contain four subclusters: sub-cluster 1a (SC1a) comprises the three samples in cuscutae semen and one sample in Japonese dodder. The sub-cluster 1b (SC1b) contains the one sample in cuscutae semen and three samples in Japanese dodder. The sub-cluster 1c (SC1c) comprises the five samples in cuscutae semen and five samples in Japonese dodder consequently. The sub-cluster 1d (SC1d) contains the two samples in cuscutae semen. But overall, thirty samples are subsequently divided into two sets: the first one contains twenty samples in cuscutae semen and Japanese dodder; the remaining samples in sinapis semen form the other group. The cuscutae semen and Japanese dodder of the first group contain similar chemical composition because they belong to the sibling plant seeds. The result does not reflect clearly the real relationship of the twenty samples of cuscutae semen and Japanese dodder in the relatives, and it does not agree with our expectations; thus, this method is not satisfactory. In order to achieve our desired results, the application of discrete wavelet analysis and RBF neural network are introduced into our study.

### 3.3. Feature Extraction of FT-IR in DWT Domain

When we use the wavelet transformation to analyze data, proper wavelet basis function and decomposing level number should be determined according to the spectral characteristics of the signal. The suitable wavelet base and wavelet scale are determined by the effect of signal decomposition in different scales and the characteristics of the FT-IR signal in wavelet multidetail decomposition procedure. There is not a general criterion about how to choose the optimal wavelet basis function. In general, we choose a proper wavelet basis function by considering the properties of the wavelet basis function, features of signal to be analyzed, and actual problem. The part of the signal whose shape is similar to that of the wavelet basis function will be enlarged, and other parts of the signal will be suppressed. In addition, proper scale wavelet is used according to the real problems. Big scale wavelet basis function should be used if we describe the total and approximate properties of the signal by the wavelet transformation. Small scale wavelet basis function should be used if we extrude the scales of the signal by the wavelet transformation. 

**Figure 4:**
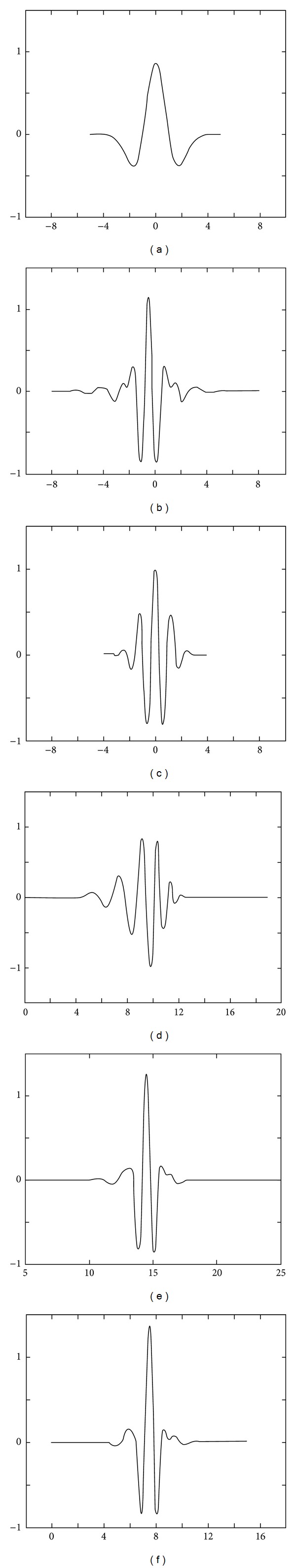
Wavelet basis function curves in time domain.

We will use the DWT to detect the singularity of the curvature curve, so we should choose proper wavelet, which has similar shape to the absorption peak analyzed, short compact branch set, and big vanishing moment, as wavelet basis function. Some representative wavelet basis functions include Mexihat, Meyer, Morlet, Daubechies, Coiflet, and Symlets. Figures 4(a)–4(f) show their function curves in time domain. Compared to other five wavelets, Daubechies wavelet has the shortest compact branch set (Figure 4(d)), so we choose Daubechies wavelet as analyzing wavelet.

**Figure 5:**
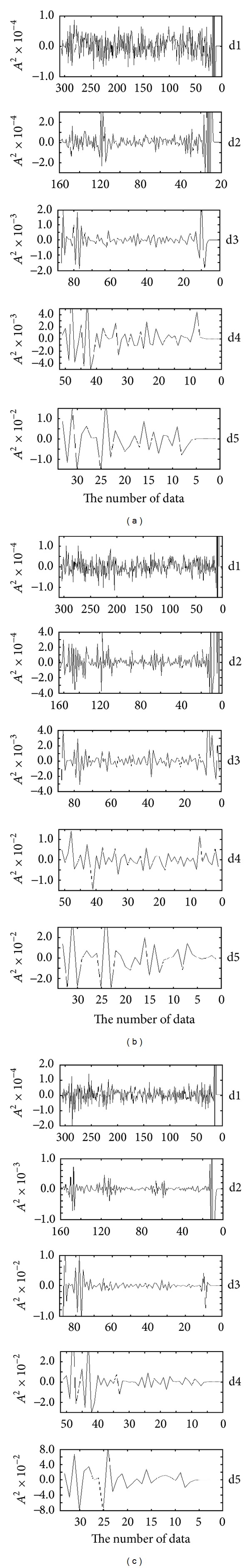
Coefficients of five scales of (a) cuscutae semen; (b) Japanese dodder; and (c) sinapis semen in DWT domain.

In this paper, the discrete wavelet transformation is done to the FT-IR spectra of cuscutae semen, Japanese dodder, and sinapis semen, respectively.  Figure 5 represents the DWT coefficients at five scales. 

 The approximation holds the low frequency components, and the detail holds high frequency components. Even the 5th scale approximation looks very similar with the original FT-IR data, but it is smoother than the original FT-IR after noise is removed. We choose representative two scales (details 3 and 4) to extract their characteristics. Characteristic variable is defined as the energy (wavelet coefficient squares) of spectrum at detail 3 and detail 4 in the DWT. 

**Figure 6:**
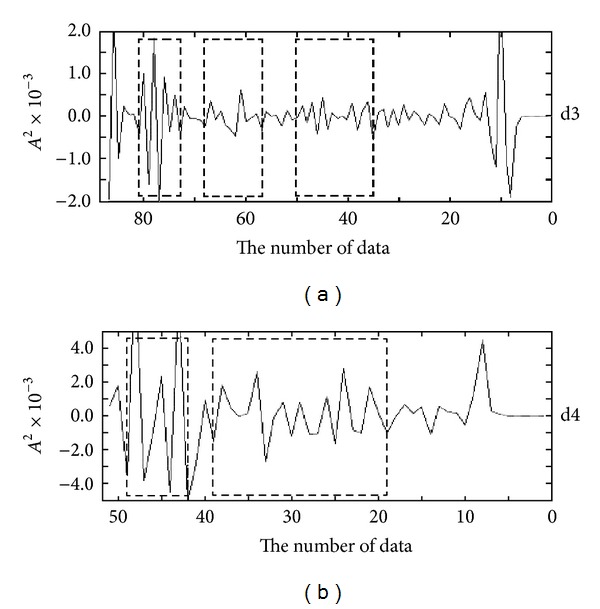
Division of feature region in the DWT domain.

According to Figure 5, the differences of DWT coefficients among the cuscutae semen, Japanese dodder, and sinapis semen are obvious in five regions. In order to effectively extract representative characteristics within two details of DWT, the spectra in each detail is divided into two and three representative regions, respectively.  Figure 6 is the division diagram of the feature regions. Five feature regions of two details in the DWT domain, whose feature values are the spectra energy in the five feature regions, form the feature vector.

### 3.4. Identified Network and Application of the Results

In order to verify the validity of proposed method, we test our method using the FT-IR spectra of 120 sets of cuscutae semen, Japanese dodder, and sinapis semen. The output layers of the RBF neural network were divided into category 1: cuscutae semen; category 2: Japanese dodder; and category 3: sinapis semen. Where 60 sets of samples are used to train RBF neural networks, and the remaining 60 sets of samples are used to test the performance of neural network.  Table 1 shows the training and testing results by RBF neural network. 

From Table 1, we can see that the identification rate with RBF neural network to identify the cuscutae semen and sinapis semen is 100%, respectively, while training samples in Linyi and testing samples in Leshan of identification rate of Japanese dodder are both 95%, respectively. So, the cuscutae semen, Japanese dodder, and sinapis semen can be correctly identified by combining RBF neural network with discrete wavelet features.

## 4. Conclusion

The application of wavelet transformation and ANN in analytical chemistry is currently a very active field of research. Direct determination of plant seed samples by HATR-FT-IR is convenient and fast. The proposed method has a high recognition rate to the cuscutae semen and its confusable derivatives Japanese dodder and sinapis semen by combining RBF neural network with the DWT features of FT-IR of samples. Wavelet transformation combined with the ANN made them attractive in many applications. Given the activity in this field, we can expect much progress in the future.

## Figures and Tables

**Table 1:** Training and testing results by RBF neural network.

	Location	Identification rate of cuscutae semen	Identification rate of Japanese dodder	Identification rate of sinapis semen
Training samples (60 sets)	Jinhua	100	100	100
	Linyi	100	95	100
	Leshan	100	100	100

Testing samples (60 sets)	Jinhua	100	100	100
	Linyi	100	100	100
	Leshan	100	95	100
